# Systematic Review of Mixed Studies on Malaria in Pregnancy: Individual, Cultural and Socioeconomic Determinants of Its Treatment and Prevention

**DOI:** 10.3390/tropicalmed7120423

**Published:** 2022-12-08

**Authors:** Jaiberth Antonio Cardona-Arias

**Affiliations:** School of Microbiology, Universidad de Antioquia UdeA, Calle 70 No. 52-21, Medellin 050010, Colombia; jaiberth.cardona@udea.edu.co

**Keywords:** malaria, qualitative research, quantitative analysis, systematic review, pregnancy, prevention, treatment

## Abstract

Malaria in pregnancy (MiP) is a global public health problem; its research is predominantly quantitative. The objective was to analyze the individual, cultural and socioeconomic determinants of the treatment and prevention of MiP with a systematic review of mixed studies (search had no date restriction). Reproducibility and evaluation of the methodological quality were guaranteed. 21 studies were included (20 from Africa). The quantitative component included 7816 pregnant women and 483 health workers. The qualitative component included 800 subjects (pregnant women, health workers, family members and community leaders). The main topics were the use and acceptability of WHO strategies to prevent MiP, individual determinants related with knowledge, perceptions, attitudes and behaviors on MiP, and cultural and socioeconomic barriers for its treatment and prevention. The main determinants of MiP were long distance to the clinic, lack of economic resources, low-coverage antenatal care, few health workers in the communities, drug shortages, cultural rules that prevent women’s participation in health issues, and misconceptions about MiP. MiP has determinants related to economic conditions, the structure and functioning of the health system, symbolic and cultural aspects, as well as knowledge, beliefs, perceptions and behavior of pregnant women, which prevent optimal access and use of preventive strategies. This study evidences the importance of intersectional, intersectoral, and interdisciplinary work to prevent MiP.

## 1. Introduction

Malaria in pregnancy (MiP) is a problem for global public health because it has epidemiological, clinical and socioeconomic impacts. Epidemiologically, in 2021 the World Health Organization (WHO) registered 241 million cases of malaria (without specifying the proportion of infected pregnant women), and WHO experts estimate more of 30 million pregnant women at risk of MiP [1,2,3]. Clinically, untreated MiP increases the risk of severe malaria, maternal and fetal anemia, premature delivery, low birth weight, and fetal, maternal and neonatal death [3]. Socioeconomically, MiP increases the costs for the health system, and the patients, by transportation to the clinic, treatments for fever, and time lost due to disease care [4].

Furthermore, there exists socioeconomic inequalities in the access and use of preventive strategies for MiP such as insecticide-treated mosquito nets (ITNs) and intermittent preventive treatment with sulfadoxine–pyrimethamine (SP-IPTp) according to economic income, level of education and residing area [5,6,7]. In general, there is a confluence between malaria and poverty with various feedback mechanisms; thus, it is necessary to investment in strategies that decrease socio-economic disparities to reduce the malaria burden [8].

WHO strategies for MiP include SP-IPTp, ITNs, indoor residual spraying (IRS), and the detection and treatment of cases [3,9], which have not achieved the expected results and the frequency of MiP remains high. A meta-analysis with 14 studies from Colombia reported a prevalence of 16.7% [10]; a meta-analysis of 16 studies from India reported 11.4% [11], and in sub-Saharan Africa frequencies up to 45.8% have been reported [12].

The high prevalence of MiP, despite the availability of effective preventive methods [13,14], could be attributed to individual, cultural or socioeconomic determinants, or barriers to the use and acceptability of preventive, diagnostic and therapeutic strategies. These determinants of MiP have been investigated mainly by qualitative studies. Some reviews of qualitative research have identified the following determinants for the success of strategies to control MiP: interpret the women’s vulnerability with local categories, understand health worker–pregnant woman interactions, analyze the household decision-making and gender relations, investigate the cost and distance to health facilities, assess healthcare infrastructure (antenatal care (ANC) services, drugs and ITNs stocks), and increase studies related to attitudes and practices of health workers, patient adherence, and beliefs of the community [15,16,17].

Qualitative evidence has helped to identify MiP determinants, but its results cannot be generalized; on the other hand, the quantitative evidence, despite being predominant in MiP and having advantages such as large sample sizes, trend analysis and result generalization, it does not delve into the contextual determinants (socioeconomic, or cultural). These limitations show the need to systematize the evidence generated with mixed studies (multi-methods) to allow the integration and discussion of quantitative and qualitative findings, transcend dualistic visions of reality, overcome the limitations to unite the strengths of each method, perform meta-inferences, broaden the understanding of the study problem, propose better solution alternatives [18,19], improve the evaluation of practices, interventions or programs of health, and articulate objective, subjective and intersubjective evidence [20,21,22].

The objective of this systematic review was to analyze the individual, cultural and socioeconomic determinants of the treatment and prevention of MiP, reported in mixed studies from the world scientific literature. For the achievement of the objective the following PICo (population or problem, interest and context) question was formulated:

Population: participants of mixed studies on MiP, including pregnant women, relatives, health workers, personnel related to malaria or ANC programs, and community leaders.

Interest: articulation of qualitative and quantitative findings on the prevention, diagnosis or treatment of MiP.

Context: research in community contexts or healthcare programs in endemic malaria areas.

## 2. Materials and Methods

### 2.1. Study Type

Systematic review of mixed studies, applying the PRISMA (preferred reporting items for systematic reviews and meta-analyses) guide [23].

### 2.2. Data Source and Searches

To identify the search terms two strategies were implemented: (i) query in Thesaurus DeCS (in Spanish Descriptores en Ciencias de la Salud) and MeSH (Medical Subject Headings), (ii) comprehensive pearl growing [24]. This led to the selection of the following terms: (i) three for the disease: malaria, *Plasmodium* and paludism; (ii) four for the study group: pregnancy, gestation, placenta and congenital, and (iii) nine for the method: mixed methods, quantitative/qualitative research, hermeneutic, ethnographies/ethnography, grounded theory, community-based participatory research/community-based research, participatory research/participatory action research, cultural anthropology and ethnopsychology.

With these terms, nine search strategies were applied in PubMed, OVID EMCare, Scielo, Scopus, Web of Science, LILACS, Science-Direct, Jstor, Campbell Collaboration/Cochrane Library, HAPI and Google Scholar (Table 1). This process was complemented with a manual search of the references of the selected manuscripts. All search strategies were also applied in Spanish and Portuguese (without finding additional results), and there was no date restriction (the search was updated in October 2022).

### 2.3. Eligibility Criteria

For the records identified with all the search strategies, the first inclusion criteria was applied: include the search terms in the title or abstract. Subsequently, the manuscripts were saved in a common source in Zotero to eliminate duplicates, and the following inclusion criteria was applied: studies on MiP as the main topic, original investigation (reviews, editorials, essays were excluded), research with mixed methods (studies exclusively quantitative or qualitative were eliminated), and studies available in full text (two articles were not available, author gave no response upon correspondence).

### 2.4. Study Selection and Data Extraction

The following variables were extracted from the included studies: title, authors, year of publication, place of study, central topic, objective, number and characteristics of the study subjects, type of mixed study, quantitative information collection instruments, qualitative research techniques, central results of the quantitative component, categories (with its properties and dimensions) of the qualitative component, articulation of the data of each approach, and conclusions.

### 2.5. Quality Assessment and Reproducibility

The researchers applied the search and selection protocol at two different times to guarantee reproducibility. The methodological rigor was evaluated with mixed methods appraisal tool (MMAT) [25] which includes five criteria to assess the quality of this type of studies. The last criteria of MMAT evaluates each method involved, because the quantitative component in all the studies was observational, the methodological quality criteria of Strengthening the reporting of observational studies in epidemiology (STROBE) [26] were chosen; while for the qualitative component the standards for reporting qualitative research (SRQR) was used [27] which is a guide with flexible and generic methodological quality criteria, applicable to all qualitative designs.

### 2.6. Data Analysis

The percentage of quality criteria fulfilled by each study was determined; to this an analysis by item (number of studies that applied each criterion) was added. The quantitative component identified several outcomes and associated factors with MiP, but only with ITN use was it possible to perform a meta-analysis (the other outcomes were reported by one or two studies, so it was not feasible to perform a meta-analysis). This meta-analysis estimated the combined proportion of ITN use, with a random effects model, due to the high heterogeneity among studies (assessed with the I^2^ inconsistency statistic); the sensitivity was assessed by the weight of each study in the combined measure, and publication bias was analyzed with the Begg statistic. The meta-analysis was performed in EPIDAT 3.1 with a confidence of 95%.

For the other variables, a synthesis was made highlighting the main quantitative and qualitative findings of each mixed study. Finally, the results were grouped into a conceptual matrix with four levels to present the meta-synthesis: (i) MiP and its consequences, (ii) WHO strategies for MiP control; (iii) individual determinants (or knowledge–opinions, perceptions, attitudes, and behavior-practices) regarding the prevention, treatment and consequences of MiP; (iv) cultural and socioeconomic determinants of MiP prevention and treatment.

## 3. Results

**Figure 1:**
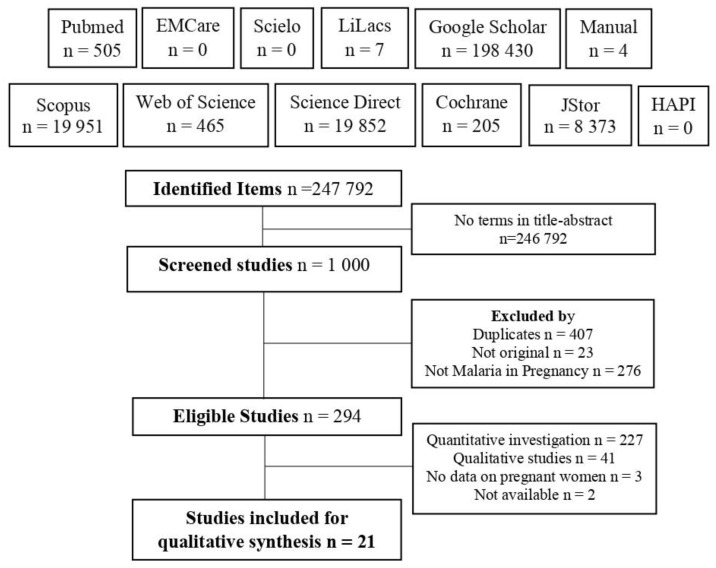
Flowchart of the search and selection of included studies.

The application of searches generated 247,792 results; however, only 21 met all the eligibility criteria (Figure 1).

### 3.1. Study Population

The studies were published between 2004 and 2022; 20 were conducted in Africa (Table 2). In the quantitative component, surveys were applied to 7816 pregnant women (mainly from rural areas) recruited in hospitals and 483 health workers; in other studies, secondary sources were used to estimate the administered doses of SP-IPTp. The qualitative component included around 800 subjects in 40 focus group discussions (with pregnant woman = 25, health workers = 11, community people = 4), 746 interviews with health workers, key informants, members of the traditional medical system, pregnant woman, women of reproductive age, and men; to this were added 59 observations from ANC services (Table 2).

### 3.2. Methodological Quality and Main Topics

**Figure 2:**
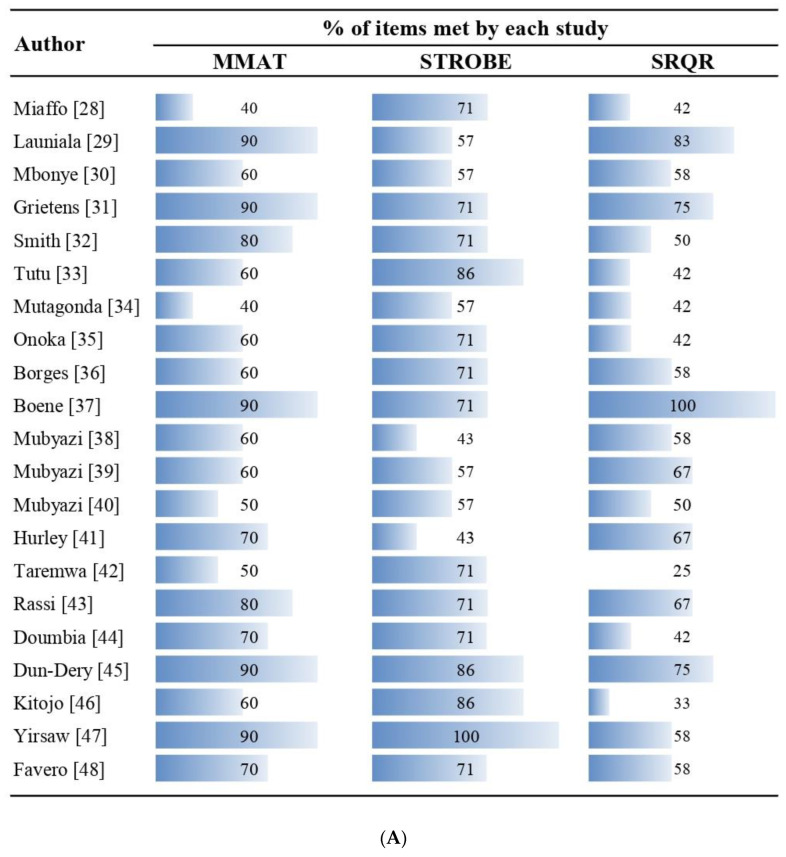

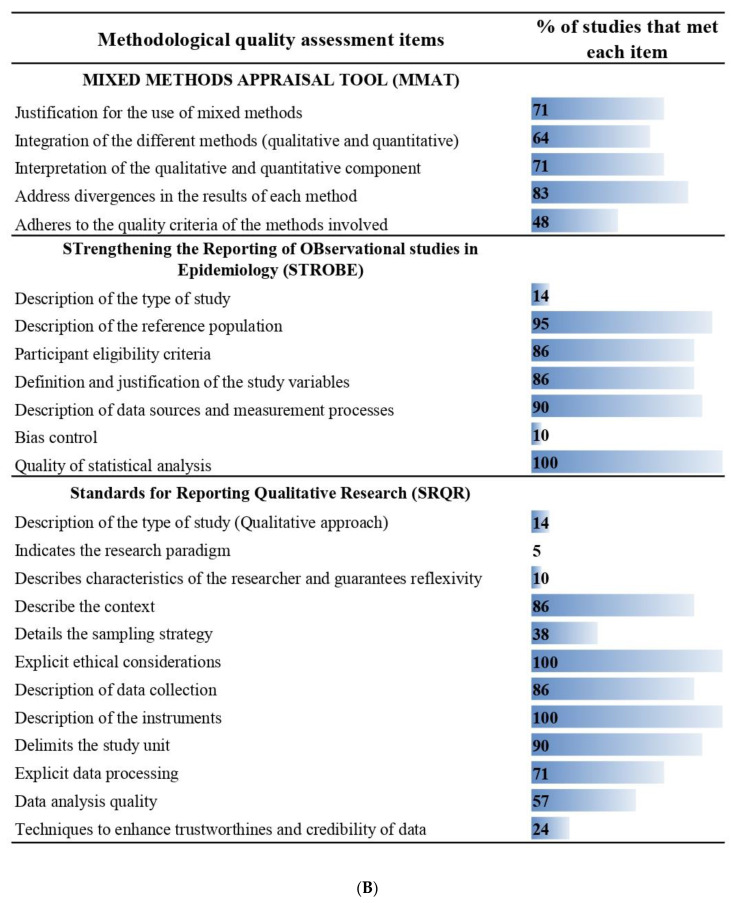
Methodological quality. (**A**) Evaluation of each study, (**B**) Evaluation of each quality item.

Ten studies met 70% or more of the MMAT criteria, in the qualitative component only four studies were above this value in the items of the SRQR guide (Figure 2A). The criteria least applied were “adheres to the quality criteria of the methods involved” of the MMAT; bias controls and description of the type of study in STROBE; while in SRQR were the indication of the research paradigm, characteristics of the research and guarantees of reflexivity, type of study, and techniques to enhance trustworthiness and credibility of the data (Figure 2B).

The largest proportion of studies did not indicated the type of mixed study carried out (they only stated the use of quantitative and qualitative techniques); a mixed convergent study was found [43], one explanatory sequential QUAN-Qual [45] and one exploratory QUAN-Qual [44]. Only two studies made explicit the qualitative approach used [29,37].

The integration of qualitative and quantitative evidence was accomplished in the following ways: the qualitative results were used to create a survey, some findings of the questionnaire were deepened in the interview, the quantitative findings were explained by the qualitative approach, barriers to adherence to a strategy at the individual level were studied quantitatively and the qualitative component was used to explain institutional and sociocultural barriers. Table 3 presents the main results of the quantitative and qualitative components.

### 3.3. WHO Strategies

#### 3.3.1. SP-IPTp: Intermittent Preventive Treatment in Pregnancy with Sulfadoxine–Pyrimethamine

SP-IPTp is effective in reducing the proportion of malaria, anemia, and maternal morbidity (malaria-related), producing few adverse effects [33]. Despite its effectiveness and safety, pregnant women prefer other interventions, such as ITN [37]; few pregnant women take the complete treatment (<30%) [31,45], and the reports of its coverage are unreliable [38,41].

Health workers have a good level of knowledge about this intervention, 88% know the SP-IPTp policy [32], 81% know the guidelines for its administration, and 83% knew that the guidelines recommend at least two doses [39]. The knowledge and use of SP-IPTp could be improved with simple interventions, as shown in the studies of Rassi [43] and Doumbia [44].

The socioeconomic, cultural, behavioral and health-system determinants of the acceptability, use and effectiveness of SP-IPTp included the following: (i) low participation of pregnant women with health issues due to internal regulations of the community [31], occupied with work or domestic tasks [31], and a high change of residence (mainly due to crops or economic issues) [45], (ii) scarce knowledge about the prescribed drugs in ANC [33,37,41], (iii) lack of knowledge about the importance of SP-IPTp [45], spread of rumors in the community about the risks to health for use of SP [39], ineffective educational campaigns not being designed with knowledge of the target audience [31], and (iv) problems contacting ANC services [45], user disappointment with waiting times [39,45], drug stock-outs, provider negligence/absenteeism, and mishandling of adverse drug reactions [45].

Health workers prefer prevention over cure [32] and they identify the effectiveness of SP-IPTp to reduce malaria and protect newborns [33]. However, they indicate that its use is hampered by the following factors: (i) scarce knowledge of the ANC staff [32,33], unknown SP-IPTp doses [39] and poor management of adverse effects [33], (ii) hospitals not administering SP-IPTp-SP through directly observed therapy, neither at correct intervals, nor free [32,41], (iii) SP-IPTp supply generating work overload [32,38,39], and (iv) pregnant women often register late at clinics and some do not keep ANC appointments regularly [39].

#### 3.3.2. Insecticide-Treated Mosquito Net (ITN)

In four studies [28,33,37,47] that surveyed 1340 pregnant women, the prevalence of ITN use was meta-analyzed, obtaining a combined measure of 49% using a random effects model (I^2^ = 0.9. Begg *p* > 0,05); this prevalence ranged from 3% in ANC non-users [28] to 77% in users of an ANC [37]. Factors associated with its use included the predominant material at home, living in a rural area, and having more than two rooms in the house [47].

ITN is the most preferred preventive strategy among pregnant women [37]. It is considered a core action of the ANC because they prevent various vector-borne diseases and protect more than one person [38]. Due to these advantages, it is recommended to improve the distribution through providers, health personnel and pregnant women [37].

Other authors reported low knowledge about the importance of ITNs, as well as barriers to its use, such as erroneous beliefs (ITNs increase heat and create bedbugs), insufficient access, lack of timely immersion of ITNs in insecticides, lack of allocation proportional to family size, ITN use for purposes different to malaria prevention, and lack of adequate places to sleep [47].

#### 3.3.3. Policy of Screening Test and Treatment

Among health workers in ANC, 20% knew the malaria treatment policy for the first trimester of pregnancy, 42% knew the recommendations for the second/third trimester [32]. Among pregnant women 97% perceived the detection programs favorably, almost all of them suggested continuing with the strategies of the Ministry of Health, with some recommendations such as expanding the type of health personnel who can deliver medicines, increase educational campaigns for health workers, and improve monitoring mechanisms of the implementation [46].

In other contexts, it has been reported that less than half (45%) of pregnant women with malaria received the first treatment recommended in the national guidelines, which is related to suboptimal knowledge of health personnel about its management, and poor connection of the program malaria with ANC [36]. Furthermore, in some places the health workers identified negative consequence of the introduction of screening because this strategy brings an additional workload for ANC providers. [32].

### 3.4. Knowledge, Perceptions and Behaviors Related to the Prevention and Treatment of MiP

The quantitative analyses highlighted the following results: about a third of the pregnant women had low knowledge on malaria [47], 74% relate malaria to the mosquito, 65% consider pregnant women as a at-risk group, 48–58% did not perceive themselves at risk of malaria [37,47], and the preference for prophylaxis with chloroquine ranged between 5% [41] and 65% [28]. For health workers malaria is the most frequent cause of maternal anemia (80%), low birth weight (70%) and premature birth (82%) [32].

From the qualitative perspective the following findings on malaria in general were highlighted: (i) malaria and anemia are perceived as diseases with high frequency [28,37]; in some communities malaria was not specifically referred to, but autochthonous terms are used for diseases that cause fever [29]; (ii) despite the high frequency of fever, it was common to consider these diseases as harmless, and pregnant women as a population with low-malaria susceptibility [29], (iii) untreated MiP increased death risk [47], (iv) the communities showed misconceptions about the disease and its prevention [36,47].

MiP qualitative approaches allowed to recognize the importance of prophylaxis [28]; health workers perceived pregnant women as a vulnerable population and MiP as an event that requires specialized medical care [36]. From the perspective of pregnant women, the greater risk of MiP is explained by the fact that the mother must share her protection with the child; there is low knowledge of the risks and adverse consequences of MiP, the majority described consequences of malaria for maternal health (anemia, weakness, loss of appetite or death), with few identifying consequences for the fetus or newborn (low birth weight, premature birth or abortion) [32,37].

### 3.5. Antenatal Care and other Structural (Sociocultural) Determinants of MiP

The coverage of at least one ANC control was 95% in villages with ANC services and 40% in villages without this service [28]. The coverage of at least three ANC control was 55% in villages with ANC services, 7% in villages without ANC [28]; 45% in adolescents (primigravidae 47% and secundigravidae 44%), and 59% in adults (primigravidae 62% and secundigravidae 55%) [31].

The reasons for late admission to ANC included long distance to the clinic, carelessness and negligence of pregnant women or their relatives, fear of being seen pregnant, domestic occupations, social position and cultural responsibilities of women, high costs (user fees, ITN voucher, transportation) and lack of economic resources, ignorance of health risks and misinformation about the benefits of ANC [28,31,38].

**Figure 3:**
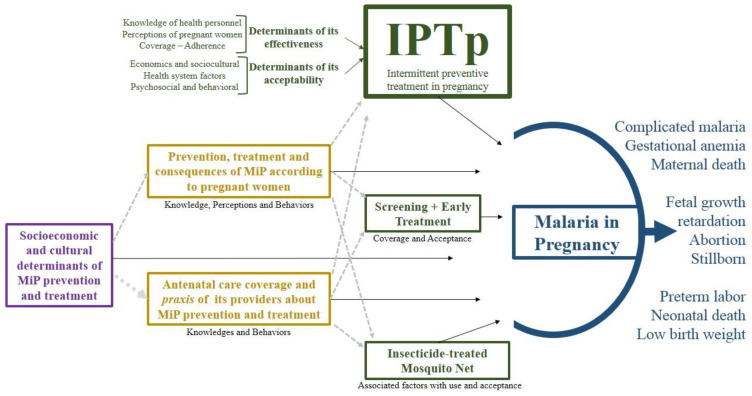
Meta-synthesis of qualitative and quantitative results.

The qualitative and quantitative evidence allowed the elaboration of a meta-synthesis in which the central themes of this review are grouped into four levels: (i) MiP and its consequences on maternal, fetal and neonatal health, (ii) coverage (use), acceptability and factors associated with the WHO strategies for MiP control (SP-IPTp, ITN and screening with early treatment); (iii) knowledge (or opinions), perceptions, attitudes and behaviors (or practices) regarding the prevention and treatment of MiP; the coverage, knowledge and behaviors related with ANC, and (iv) socioeconomic and cultural determinants of prevention and treatment. The meta-synthesis highlights SP-IPTp as this was the central topic of most of the systematized studies (Figure 3).

## 4. Discussion

### 4.1. Populations, Methodological Quality and Mail Topics of Systematized Studies

The findings show several important issues:

1. The advantages of systematic reviews, such as achieve greater possibilities of the extrapolation of the results, increase the statistical power and precision, group the published evidence of this topic, and identify and relate the central categories of qualitative evidence, among others [49].
2. The concentration of evidence in Africa shows that MiP research is incipient in other continents, as has been documented by a previous review [50].
3. Despite the relevance of mixed methods, they are marginal in malaria research, other researchers have reported that this area has been hegemonically positivist [15], which should warn about the dimensions and determinants that are not cognizable by quantitative components, and derive recommendations that will only achieve partial control of MiP.

In encouraging the development of mixed studies, investigators, funders, reviewers, journal editors, and other stakeholders should improve the methodological rigor, especially in critical items as the type of mixed study, bias control, type of quantitative and qualitative study, research paradigm, reflexivity, trustworthiness and credibility.

The meta-synthesis allowed to relate the prevention and treatment strategies with the individual, cultural and socioeconomic determinants, as well as establish some paths and mechanisms that produce MiP. This was made possible by the combination of the advantages of the qualitative (deep understanding of the meanings, attitudes, behaviors, interactions, and social processes of daily life) and quantitative (measure and relate variables, establish trends, make predictions, explanatory models and generalizations with a large number of participants) approaches [18,19,20,21,22].

This meta-synthesis presents several interesting implications: (i) identify successful determinants of the WHO strategies, (ii) relate categories that explain the persistence of MiP in some territories, (iii) make explicit some determinants that should be included in the control policies, (iv) determine the key contents of health communications for pregnant women and ANC workers, (v) describe some determinants of prevention and treatment of MiP in the African context, which should become the objective of research in other continents.

### 4.2. SP-IPTp

SP-IPTp reduces malaria and anemia, which coincides with previous studies that have shown its efficacy [13]. However, this intervention it is not included in the preferences of pregnant women, nor meets the expected coverage as few pregnant women take the complete treatment. Triangulating qualitative and quantitative techniques possibly explained this situation with three types of determinants: of the health-system, of the community and of pregnant woman.

The determinants of the health system that explain the low use of SP-IPTp include low-coverage ANC, work overload, negligence or absenteeism of providers, long waiting times, drug shortages and educational campaigns without knowledge of the target group. The magnitude of these problems is serious, as demonstrated by others studies with the following findings: (i) data from the Demographic and Health Surveys 2015–2020 in sub-Saharan Africa, the analyses of 113,918 pregnant women showed an overall health insurance coverage of 4.4% and timely ANC attendance of 39.0% [51]; (ii) in sub-Saharan Africa ANC has structural barriers, such as lack of funding, infrastructure, distribution and human resource [52], (iii) South Africa faces serious problems of affordability, availability and distribution of its health workforce [53], and (iv) other reviews have concluded that the provision and uptake of SP-IPTp is affected by human resource shortages, drug stock-outs, conflicts on free provisions, hidden costs, and poor quality of care [54]. In relation to the design and implementation of educational strategies, it should be noted that their effectiveness depends on their adjustment to local realities; as demonstrated in a study on health education based on information-motivation-behavioral skills, which improved ITN use and SP-IPTp uptake [55].

The community determinants include cultural rules that prevent women’s participation in health issues (their focus is domestic tasks), and rumors and misconceptions about risks of the use SP. The understanding, explanation and intervention of these aspects is too complex given that in the hegemonic public health and in the biomedical perspectives the center is this disease with scarce research on the illness and sickness dimensions. The terms disease, illness and sickness allude different and complementary dimensions for healthcare; however, they are studied separately [56]. Further MiP research must be conducted to capture the essence and normative implications of these concepts and integrate theoretical and empirical perspectives with patients’ views and their sociocultural determinants [55].

The individual determinants of MiP were low knowledge about the importance of SP-IPTp, register late at clinics and not keep ANC appointments. These determinants are susceptible to intervention with short-term strategies, such as health education [55]. Although it should be noted that the success of these interventions also depends on community determinants, symbolic-cultural aspects and the type of health system, which should be taken into account in all health actions, such as medium- to long-term strategies that have been proposed in others meta-analyses about the delivery, access, and use of interventions to prevent MiP [7].

### 4.3. ITN

In this systematic review ITN was the most preferred preventive strategy among pregnant women, the prevalence of use was 49%; the factors associated were the material at home, living in a rural area, and having more than two rooms. These data differ from previous meta-analyses on ITNs that reported the possession of 75.8% and use of 58.3% [57]; although it should be noted that other meta-analyses concluded that self-reporting overestimated ITN adherence in comparison to objectively measured ITN use [58].

Divergences were also found in the associated factors, given that other systematic reviews on ITN have reported the wealth quintile, the number of children under 5 in the household, the education level of the head of household, and the knowledge that sleeping under a mosquito net protects against malaria [58], demonstrating the need of more studies on this theme.

In this meta-synthesis the main barriers to ITN use were low knowledge, insufficient access, lack of allocation proportional to family size, and lack of adequate places to sleep; factors that are integrated into the findings of other systematic reviews [7,15,57,58], demonstrating the complexity of the determinants of MiP prevention, since it includes economic, sociocultural, community, housing and individual aspects.

It is important overcome these barriers for the following reasons: it is the alternative preferred by pregnant women, its handing over in ANC presents fewer problems [7], and is the most effective for malaria prevention, as shown by a meta-analysis that reported the following relative risk of presenting malaria with different strategies: ITN = 0.49 (95% CI: 0.32–0.74); prophylactic drugs = 0.24 (95% CI: 0.004–15.43), indoor residual spraying = 0.55 (95% CI: 0.20–1.56) and untreated net = 0.73 (95% CI: 0.28–1.90) [59].

### 4.4. Policies or Actions of the Health System related to Antenatal Care

All pregnant women had favorable perceptions of the ANC and MiP programs; to improve the malaria control policy the following negative aspects must be tackled: monitoring mechanisms, knowledge of health personnel, workload for ANC providers and connection among ANC and malaria program. In this component of the meta-synthesis, it is interesting to know that both, the quantitative and qualitative approaches, showed the centrality of factors related with the health system, which reiterates the importance of investigating structural issues of this domain, such as the coverage of ANC, the availability of human resources in healthcare, and other factors previously discussed [51,52,53].

### 4.5. Knowledge, Perceptions and Behaviors related to the Prevention, Treatment and Consequences of MiP

In this component the mixed studies reported low knowledge about MiP and its consequences, low perceptions of risk, and allusion to malaria as a topic of community interest that demands action of the district authorities. Contrary to the previous category, in this topic only individual determinant were identified, which can be effectively impacted with short-term interventions [55].

Despite the possibilities of intervening in these factors through educational actions, it is worrying to know that pregnant women in endemic territories do not have sufficient knowledge about the risks and adverse effects of MiP, and simultaneously delegate the responsibility for its control to the district health authorities. In a meta-synthesis of qualitative studies on malaria in Colombia, a different situation was found, describing that “they show similar qualitative evidence on structural determiners, family-individual effects, and ways to understand malaria”; however, the Colombian research presents a recommendation applicable to what was found in this synthesis of mixed studies: “motivations to participate in disease interventions are less known, and they constitute the central axis for subsequent studies aimed to improve community engagement in disease control” [60].

The low knowledge of pregnant women converges with the conviction that MiP is a topic of community interest that demands action of the district authorities, without alluding to individual responsibilities. This kind of paternalism has been the subject of several reflections in other areas such as mental health and bioethics. Some consider that weak paternalism in healthcare can optimize preventive and therapeutic actions [61], and others have criticized paternalism in healthcare given that it exceeds the limits of social protection and annuls autonomy [62,63]. Intermediate positions criticize strong paternalism, but soft paternalism is always desirable in healthcare in several aspects: expert recommendations on treatment, recognition of the importance of clinical experience, promotion of healthy behaviors, control demands for healthcare that can be expensive and unnecessary [64]. Despite these discussions, the findings of this meta-synthesis show the need to recognize and intervene in individual, cultural and socioeconomic determinants, which advocates a balance between social protection programs and personal responsibility with the care of one’s own health.

### 4.6. Antenatal Care and other Structural Determinants of MiP

In this component, the mixed studies reported main determinants of MiP as low coverage of ANC, attributable to economic and individual factors. The economic factors included long distance to the clinic, lack of economic resources and high costs of healthcare. This converges with other studies that have reported low coverage of health insurance and timely ANC attendance [51], high costs of MiP attention for health systems and patients [4], socioeconomic inequalities in the use of ITNs and SP-IPTp according to economic income, level of education and residing area [5,6,7].

The individual factors included carelessness and negligence of pregnant women or their relatives, and ignorance of health benefits of ANC. This is worrying, not only for the treatment and prevention of MiP, but for all the foreseeable events during pregnancy. It is also a negative finding given multiple reported evidence for the benefits of ANC to reduce low birth weight, pre-term birth, perinatal mortality, and other outcomes [65,66,67].

### 4.7. Limitations

The studies focused on the perspectives of pregnant women and health workers, without actors related to the implementation and evaluation of public policies on malaria. The studies addressed the WHO strategies (SP-IPTp, ITNs, screening) with few studies addressing the determinants of treatment and prevention as the central outcome, these emerged as secondary factors that require further investigation. One last limitation was the concentration of evidence in Africa, with the absence of mixed evidence from other continents.

### 4.8. Strengths

This meta-synthesis included a large sample size of pregnant women and health workers. It is the first manuscript that summarizes the mixed evidence available, which is important for MiP research and mixed methods in healthcare. This research articulates that evidence on MiP is generally scattered and conducted with a single approach, which does not capture the complexity of MiP. The meta-synthesis identified determinants of MiP and of the WHO strategies, which allows establishing lines of research to prevent MiP and its consequences.

### 4.9. Contribution to Public Health Policies

This research identified factors of the economic system, health-system, community, housing and of pregnant woman, which should be included in the design, implementation, and evaluation of public health policies for MiP. Health policies aimed at MiP must include actions and strategies that balance (harmonize) social protection with personal responsibility in health care.

Health policies for MiP control should include: (i) strategies to increase ANC coverage and the use of WHO strategies for MiP; (ii) a social work team to overcome cultural barriers, (iii) health educators to improve KAP on MiP of pregnant women and health personnel, (iv) assertive and effective communication in healthcare to correct misconceptions about biomedical interventions, (v) articulation with care of other febrile diseases during pregnancy, (vi) an economic axis to subsidize or make direct transfers aimed at correcting the economic barriers of actions for the prevention, diagnosis and treatment of MiP, and thereby reduce out-of-pocket spending on health.

## 5. Conclusions

Prevention and treatment of MiP, focused on traditional strategies such as epidemiological surveillance, screening and use of SP-IPTp and ITNs, will not achieve the control and elimination goals, since MiP has other determinants related to economic conditions, the structure and functioning of the health system, symbolic and cultural aspects of the affected communities, as well as knowledge, beliefs, perceptions and behaviors of pregnant women. Only research based on mixed methods can allow for identification and intervene adequately the complexity of these determinants. This review demonstrates the importance of intersectional, intersectoral, and interdisciplinary work to prevent MiP, with short-, medium- and long-term research to impact the objective, subjective, and intersubjective aspects related to MiP.

## Figures and Tables

**Table 1:** Search syntax applied to database.

Source	Search Syntax
**PubMed**	((Malaria[Title/Abstract] OR Paludism[Title/Abstract] OR Plasmodium[Title/Abstract]) AND (Pregnancy[Title/Abstract] OR Gestation[Title/Abstract] OR Placenta[Title/Abstract] OR Congenital[Title/Abstract])) AND (Mixed methods[Title/Abstract])
	((Malaria[Title/Abstract] OR Paludism[Title/Abstract] OR Plasmodium[Title/Abstract]) AND (Pregnancy[Title/Abstract] OR Gestation[Title/Abstract] OR Placenta[Title/Abstract] OR Congenital[Title/Abstract])) AND (qualitative[Title/Abstract] OR quantitative[Title/Abstract])
	((Malaria[Title/Abstract] OR Paludism[Title/Abstract] OR Plasmodium[Title/Abstract]) AND (Pregnancy[Title/Abstract] OR Gestation[Title/Abstract] OR Placenta[Title/Abstract] OR Congenital[Title/Abstract])) AND (hermeneutic[Title/Abstract])
	((Malaria[Title/Abstract] OR Paludism[Title/Abstract] OR Plasmodium[Title/Abstract]) AND (Pregnancy[Title/Abstract] OR Gestation[Title/Abstract] OR Placenta[Title/Abstract] OR Congenital[Title/Abstract])) AND (ethnographies[Title/Abstract] OR ethnography [Title/Abstract])
	((Malaria[Title/Abstract] OR Paludism[Title/Abstract] OR Plasmodium[Title/Abstract]) AND (Pregnancy[Title/Abstract] OR Gestation[Title/Abstract] OR Placenta[Title/Abstract] OR Congenital[Title/Abstract])) AND (grounded theory[Title/Abstract])
	((Malaria[Title/Abstract] OR Paludism[Title/Abstract] OR Plasmodium[Title/Abstract]) AND (Pregnancy[Title/Abstract] OR Gestation[Title/Abstract] OR Placenta[Title/Abstract] OR Congenital[Title/Abstract])) AND (community-based participatory research[Title/Abstract] OR community-based research[Title/Abstract])
	((Malaria[Title/Abstract] OR Paludism[Title/Abstract] OR Plasmodium[Title/Abstract]) AND (Pregnancy[Title/Abstract] OR Gestation[Title/Abstract] OR Placenta[Title/Abstract] OR Congenital[Title/Abstract])) AND (participatory research[Title/Abstract] OR participatory action research[Title/Abstract])
	((Malaria[Title/Abstract] OR Paludism[Title/Abstract] OR Plasmodium[Title/Abstract]) AND (Pregnancy[Title/Abstract] OR Gestation[Title/Abstract] OR Placenta[Title/Abstract] OR Congenital[Title/Abstract])) AND (ethnopsychology[Title/Abstract])
	((Malaria[Title/Abstract] OR Paludism[Title/Abstract] OR Plasmodium[Title/Abstract]) AND (Pregnancy[Title/Abstract] OR Gestation[Title/Abstract] OR Placenta[Title/Abstract] OR Congenital[Title/Abstract])) AND (cultural anthropology[Title/Abstract])
**OVID EMCare ^1^**	((Malaria[Title/Abstract] OR Paludism[Title/Abstract] OR Plasmodium[Title/Abstract]) AND (Pregnancy[Title/Abstract] OR Gestation[Title/Abstract] OR Placenta[Title/Abstract] OR Congenital[Title/Abstract])) AND (Mixed methods[Title/Abstract])
**Scielo ^1^**	(ab:(Malaria OR Plasmodium OR Paludism)) AND (ab:((Pregnancy OR Gestation OR Placenta OR Congenital)) AND (ab:(mixed methods))
**Scopus ^1^**	TITLE-ABS-KEY (((malaria OR paludism OR plasmodium) AND (pregnancy OR gestation OR placenta OR congenital) AND (mixed methods)))
**Web of Science ^1^**	((Malaria OR Paludism OR Plasmodium) AND (Pregnancy OR Gestation OR Placenta OR Congenital) AND (Mixed methods)) (Abstract)
**LILLACS ^1^**	(ab:(Malaria OR Plasmodium OR Paludism)) AND (ab:((Pregnancy OR Gestation OR Placenta OR Congenital)) AND (ab:(mixed methods))
**Science-Direct ^1^**	(Title, abstract, keywords: (malaria OR Plasmodium OR Paludism) AND (Pregnancy OR Gestation OR Placenta OR Congenital) AND (mixed methods))
**Jstor ^1^**	(ab:(Malaria OR Plasmodium OR Paludism)) AND (ab:((Pregnancy OR Gestation OR Placenta OR Congenital)) AND (ab:(mixed methods))
**Cochrane Library ^1^**	(Malaria OR Plasmodium OR Paludism in Title Abstract Keyword) AND (Pregnancy OR Gestation OR Placenta OR Congenital in Title Abstract Keyword) AND (mixed methods in Title Abstract Keyword)
**HAPI ^1^**	Title: Malaria OR Plasmodium OR Paludism (and) Title: Pregnancy OR Gestation OR Placenta OR Congenital (and) Title: mixed methods
**Google Scholar ^1^**	Title: (Malaria OR Paludism OR Plasmodium) AND (Pregnancy OR Gestation OR Placenta OR Congenital) AND (mixed methods)

^1^ The search is presented with the term “mixed methods” to show the search strategy used in each database; in the remaining eight searches, the same strategy was applied, changing “mixed methods” for the other terms that refer to the method, as illustrated for PubMed.

**Table 2:** Description of the included studies according to the country and population.

Author	Year ^a^	Country	Population	
			Quantitative Component	Qualitative Component
Miaffo [28]	2004 (2003)	Burkina Faso	225 pregnant women attended in ANC (four near the village and four more than 5 km away)	Two FGD with pregnant women of ANC, two with husbands of ANC users, and two with pregnant women who do not use ANC. IDI to four health workers, seven traditional birth attendants, and 29 women community leaders
Launiala [29]	2006 (2002)	Malawi	189 pregnant women and 48 health workers	IDI to 34 women in reproductive age, four traditional advisors, two midwives, one traditional healer and two men
Mbonye [30]	2007 (2003–2005)	Uganda	1321 women who received SP-IPTp	IDI to 108 women. 60 IDI with human resources personnel, health workers and opinion leaders
Grietens [31]	2010 (2003–2006)	Burkina Faso	721 pregnant women from hospitals that promote SP-IPTp. 793 without SP-IPTp and 726 promoting chloroquine	IDI to 48 health workers and 35 key informants from the community. four FGD with 12 community health promoters, nine FGD with 32 pregnant women and family members
Smith [32]	2011 (2009)	Ghana	134 ANC Providers	IDI to 14 midwives and nurses
Tutu [33]	2011 (2006–2007)	Ghana	306 pregnant women with SP-IPTp	IDI to 17 health workers and four FGD with pregnant women (without “n”)
Mutagonda [34]	2012 (2010–2011)	Tanzania	470 pregnant women of ANC	FGD with 46 pregnant women
Onoka [35]	2012 (2010)	Nigeria	1307 women who gave birth up to one year before the study, 146 women attending ANC	Four FGD each one with 8–10 women
Borges [36]	2013 (2007–2008)	Brazil	250 medical records of pregnant women	IDI to 51 health workers
Boene [37]	2014 (2010)	Mozambique	85 pregnant women	IDI to 85 pregnant women and 30 observations in ANC
Mubyazi [38]	2014 (2003–2007)	Tanzania	Number of doses of SP-IPTp delivered in 3 years	IDI to program user (without “n”)
Mubyazi [39]	2014 (2006)	Tanzania	78 health workers of ANC	FGD with administrators, nurses, midwives and auxiliaries (without “n”)
Mubyazi [40]	2015 (2006)	Tanzania	820 ANC clients, health workers and mothers	FGD with pregnant women and health worker (without “n”)
Hurley [41]	2016 (2012–2013)	Mali	Mali Sociodemographic and Health Survey 2012–2013, Maternal Health Database	IDI to 15 pregnant women, four midwives, three pharmacists, four doctors, one community leader, two community volunteers, one mayor, one NGO member and six district health officers. FGD with eight young women, three teachers, five community volunteers, five husbands, two community leaders, two pregnant women and one health group leader. 29 observations in ANC
Taremwa [42]	2017 (2015)	Uganda	369 mothers of children under 5 years of age, and pregnant women	IDI to 15 key subject (local council leaders, district health inspector, religious leaders, health workers and members of village health teams)
Rassi [43]	2018 (2015)	Uganda	90 health workers	Four FGD (without “n”) with health workers. Three IDI to district health officers
Doumbia [44]	2021 (2018)	Mali	200 pregnant women	IDI to gynecologists (without “n”)
Dun-Dery [45]	2021 (2018–2019)	Ghana	697 pregnant women. 74 nurses and midwives	Three FGD with pregnant women (without “n”)
Kitojo [46]	2021 (2018)	Tanzania	143 pregnant women	Interview with 16 health workers (mostly nurses) of ANC
Yirsaw [47]	2021 (2020)	Ethiopia	724 pregnant women	FGD and IDI to 37 people who work on women’s health issues
Favero [48]	2022 (2017)	Madagascar	31 health providers	FGD and IDI with five community health workers, 102 caregivers and 90 pregnant women

^a^ Year of publication (year of research). ANC: antenatal care. MiP: malaria in pregnancy. SP-IPTp: intermittent preventive treatment in pregnancy with sulfadoxine–pyrimethamine. FGD: focus group discussion. IDI: in-depth interview.

**Table 3:** Main results of the included studies.

Author	Quantitative Result	Qualitative Result
Miaffo [28]	At least one visit to ANC 71%, at least three visits 28%. Use of chloroquine 65% and ITN 17%	Importance of MiP and chloroquine prophylaxis are recognized. Barriers to MiP prevention: distance from the health center, lack of economic resources and ignorance
Launiala [29]	Limited knowledge without differentiating malaria from other causes of fever, only 6% thought that malaria is common in pregnancy. Main effects of MiP: abortion (28%), maternal death (12%), anemia (11%), weakness (7%), and premature delivery (6%)	There is no term for MiP, they used a local term malungo that refers to diseases that cause fever. Most of the women did not perceive malungo as a serious illness, they considered anemia, sexually transmitted diseases or cholera more important
Mbonye [30]	The community approach increased access and compliance of SP-IPTp compared to the health-unit approach. Community approach increased pregnant women with two doses (67.5% of compared to 39.9% in health units) and threefold ITN use	Factors influencing acceptability and use of SP-IPTp: trust in community health personnel, home visits, support from spouses, education about the dangers of MiP and the benefits of SP-IPTp
Grietens [31]	58.5% with MiP by *P. falciparum*, higher risk of infection in the younger; 51% completed the recommendation of ≥3 ANC	Low-use of SP-IPTp because health education is not aimed at adolescents, pregnancies are socially hidden, internal regulations of authority limit participation of teenagers, and in the rainy season domestic work increases
Smith [32]	88.1% of providers were aware of all elements of the SP-IPTp policy, compared to 20.1% and 41.8% who were aware of the malaria treatment policy in the first or second/third trimester, respectively. Workshop attendance was a predictor of knowledge on MiP	There is a preference for prevention over cure, increased workload is a barrier to policies implementation. Health of pregnant women is a strong motivation for ANC providers. It is necessary improve the knowledge and practices of ANC staff
Tutu [33]	SP-IPTp decreased malaria, anemia and maternal morbidity, with few adverse effects. ITN use 56.5%, 24% use traditional medicine for febrile symptoms	Health workers with low knowledge on SP-IPTp, pregnant women consume drugs without knowing what they are prescribed for. Vendors do not recognize adverse effects of SP-IPTp
Mutagonda [34]	54.3% of pregnant women were unaware of SP-IPTp; 9.1%reported having had MiP. The antimalarials used by pregnant women were quinine 42.9%, SP 23.8%, artemether-lumefantrine 21.4%, and sulfamethoxyprazinepyrimethamine 2.4%. 98.3% perceived artemether–lumefantrine as an unsafe drug during pregnancy.	The study did not develop qualitative categories, some testimonies are taken to support quantitative findings
Onoka [35]	SP-IPTp coverage for the first and second doses was 13.7% and 7.3%, respectively. Among the women who could have received SP-IPTp only 14% were offered the first dose of those 99% took the drug	Pregnant women use drugs recommended by medical personnel because they believe they should be safe. ANC attendance and perceptions of side effects do not explain the low coverage of SP-IPTp
Borges [36]	Only 6.8% had malaria tests. For *P. falciparum* only 44.8% received the recommended first-line therapy; 10.2% with treatments that are not part of the national guidelines	Knowledge on MiP is suboptimal. Health workers perceive pregnant women as cooperative patients, and MiP as an event that requires specialized medical care
Boene [37]	74% associated MiP with the mosquito; 65% consider pregnant women as the highest risk group; 58% do not self-perceive at risk of malaria; 75% sleep in ITN	Participants are unaware of adverse outcomes of MiP. Most describe consequences of malaria for maternal health, few name consequences for the fetus and newborn. Medications provided in ANC serve to prevent diseases, but they do not differentiate their uses for specific problems.
Mubyazi [38]	It shows the number and coverage of 1st and 2^nd^ doses of SP-IPTp in three years. It compares SP-IPTp coverage estimation methods	A reporting system is proposed to improve shortcomings. Lacks reporting standardization, handling of lost data, and variations in the reporting system affect the coverage estimation methods
Mubyazi [39]	Knowledges on SP-IPTp: the guide recommends at least two doses (83%), is part of ANC (88%), is prevention and not treatment (55%). Barriers: fear of being seen in pregnancy (54%), long distance to the clinic (78%), carelessness and negligence of pregnant women (69%), ignorance of health risks (28%), cost (user fees, ITN voucher redemption, and transportation) (31%), domestic or community occupations (45%)	Health workers did not consider appropriate that private ANC clinics provide SP-IPTp free, because they recover costs elsewhere. Pregnant women often register late at clinics, and some do not keep appointments regularly, miss out on SP-IPTp and others ANC services. Rumors about the health risks and failures of SP, coupled with client disappointment with waiting times, limit acceptance of the SP-IPTp
Mubyazi [40]	Seeking of ANC was influenced by motivation for safe pregnancy and childbirth, and not necessarily by SP-IPTp. The main barriers for ANC are sociocultural values that stigmatize and discriminate to the pregnant women, hostile attitudes of health service providers, shortage of medicines, fees in health facilities, and pregnant women’s unawareness about ANC services.	The study does not develop qualitative categories, some testimonies are taken to support quantitative findings
Hurley [41]	SP-IPTp coverage is misleading due to their reliance on a variable (“IPTp source”) that is missing 62% of the data. In the survey of pregnant women, 56.2% take at least one dose of SP-IPTp; 5.2% chloroquine, and 1.9% another medication to prevent MiP. Most of the women who did not receive SP-IPTp were women who did not attend ANC	Many health centers do not administer SP-IPTp by directly observed therapy, neither at monthly intervals, nor free of charge. Women generally reported that SP-IPTp was available and tolerable, but were often unable to identify its name or purpose, which could affect the accuracy of responses in household surveys
Taremwa [42]	98.1% considered ITNs as a key strategy for malaria prevention. ITN possession was 84.0%, of which 66.1% used them systematically; 39% did not have a positive attitude towards ITNs.	The qualitative categories were: knowledge about malaria (caused by mosquito bites), attitude towards the use of ITN (agreeing that use ITN use helps to prevent malaria), not making effective use of ITN despite know its benefits
Rassi [43]	Intervention improve knowledge of SP-IPTp and coverage of three doses of SP-IPTp	Intervention is a feasible, acceptable and cost-effective. The text messages served as reminders for those who had attended the classroom training and helped spread information to those who did not
Doumbia [44]	After a visit to the gynecologist increased the level of knowledge and preventive actions; 83% of participants were unaware of malaria before use of the checklist vs. 15% after. Supervised SP-IPTp coverage increased from 0 to 59% after the introduction of the checklist	The intervention was effective and easy to adopt. Gynecologists recommend the use of this checklist during routine practice and generalize it to others health providers
Dun-Dery [45]	26.4% took the third dose of SP-IPTp. SP-IPTp uptake was associated with the number of maternal contacts in ANC and the gestational age	The main challenges to uptake of SP-IPTp were missed ANC contacts, knowledge gaps among pregnant women about the importance of SP-IPTp, drug stock-outs, provider neglect/absenteeism, adverse drug reactions, and change of residence
Kitojo [46]	97% had a favorable perception of the screening; 95% satisfied with the service; 99% would recommend continuing with the ministry’s strategy; 76% experienced pain and 16% anxiety in taking a blood sample for diagnosis	Service providers consider the screening and treatment policy favorable; the main challenge is that nurses cannot prescribe antimalarials. Health workers had a good understanding of the policy. The policy is not a burden because the malaria test is integrated into the routine laboratory tests of ANC
Yirsaw [47]	The prevalence of ITN use was 56.5%, associated with an iron roof in the house, rural residence, ≥ 2 rooms in the house and a high perception of barriers; 27.9% with low knowledge about MiP and ITN; 51.5% with low perception of susceptibility to malaria; 96.1% consider that ITN prevents malaria and 39.5% did not sleep under ITN	The most common individual-level barriers were related to misconceptions about ITN (increase heat and create bed bugs). Barriers at the institutional level: insufficient access, lack of timely immersion of ITNs in insecticides, lack of proportional allocation to family size, and lack of priority of vulnerable groups. Socio-cultural barriers: using ITNs for purposes other than malaria prevention, lack of adequate places to sleep, and erroneous cultural beliefs
Favero [48]	It presents costs associated with malaria case management, by type of healthcare provider	Care-seeking for fever is delayed until the ill person does not respond to home treatment or symptoms become severe. Care-seeking determinants for MiP included cost, travel time, distance, and perceived quality of care at clinics. Providers felt that the lack of basic products and workloads hampered their ability to provide MiP care services. Health community staff were not generally consulted for malaria care

ANC: antenatal care. MiP: malaria in pregnancy. SP-IPTp: intermittent preventive treatment in pregnancy with sulfadoxine–pyrimethamine. ITN: insecticide-treated mosquito net.

## Data Availability

Not applicable.
